# Analysis of galloping response in ice-coated transmission conductors under wind loads

**DOI:** 10.1371/journal.pone.0335594

**Published:** 2025-10-31

**Authors:** Shuang Wu, Haiqing Liu, Yunlong Wang, Yuxin Wang

**Affiliations:** 1 School of Civil Engineering, Liaoning Technical University, Fuxin, China; 2 Xinjiang Institute of Engineering, Urumqi, China; IGDTUW: Indira Gandhi Delhi Technical University for Women, INDIA

## Abstract

Conductor galloping is a low-frequency, large-amplitude vibration phenomenon induced by the combined action of ice accretion and wind loads, which poses severe threats to power grid safety. For 500 kV transmission lines in Xinjiang, a strain-displacement relationship based on elastic catenary theory was developed, establishing a nonlinear dynamic model for galloping of ice-coated bundled conductors with torsional stiffness. Employing D-shaped, fan-shaped, and crescent-shaped ice-accreted bundle conductors, this work investigates the influence of wake effects and wind attack angles on aerodynamic characteristics at varying spanwise lengths. Subsequently, aerodynamic loads were computed based on the derived aerodynamic coefficients, enabling galloping response analysis of ice-accreted bundle conductors. The results demonstrate that the proposed methodology facilitates efficient analysis of galloping amplitude in ice-accreted transmission conductors, and the three-dimensional numerical model significantly improves the computational accuracy of aerodynamic forces on bundle conductors. The flow field around ice-coated bundled conductors exhibits pronounced periodicity, governed collectively by wake effects, wind attack angle, ice accretion geometry, ice thickness, and wind velocity. Additionally, spacer bars with articulated connections suppress conductor galloping more effectively.

## 1. Introduction

Overhead transmission lines are essential components of power systems. Harsh weather in mountainous regions frequently leads to ice accretion on overhead transmission lines during winter, and the resulting uneven ice accumulation severely damages the safe operation of power grids. In early 2024, severe weather including low temperatures, rain and snow occurred in the Tekes region of Xinjiang, China, resulting in extensive ice accretion on overhead transmission lines. Ice-accreted conductors subjected to aerodynamic instability may result in short circuits, conductor breakage, metal fittings damage, cross-arm fatigue failure, and even collapse of transmission structures, leading to a severe threat to the normal production and life of the people [1–3]. Over the past several decades, scholars worldwide have conducted extensive research on the aerodynamic characteristics of ice-accreted transmission lines, including field measurements, experimental studies, and theoretical analyses [4–6].Nevertheless, due to the complexity and randomness of ice accretion, icing accidents on transmission lines caused by frequent extreme weather persist as problems. The mechanisms underlying transmission line icing disasters and their prevention methods remain unresolved challenges in both scientific research and engineering practice.

Extensive research has been conducted on the galloping mechanism of transmission lines in recent decades [7,8]. The aerodynamic coefficients of ice-coated conductors under wind-induced vibration and their variation patterns with angle of attack constitute critical factors in conductor galloping. The aerodynamic characteristics of ice-coated conductors have been extensively investigated through numerous experimental and numerical analyses. As early as 1981, Nigol et al. [9] conducted wind tunnel tests on ice-coated quad-bundled conductors, demonstrating that torsional aerodynamic damping significantly influences conductor vibration behavior. This discovery marked the inception of the torsional galloping mechanism theory. Building upon this foundation, Keutgen et al. [10] measured the aerodynamic coefficients of conductors using actual ice accretion profiles in wind tunnel tests. Their findings demonstrated that D-shaped ice-coated conductors exhibit significantly higher susceptibility to galloping excitation than crescent-shaped or fan-shaped ice-coated conductors. In addition to wind tunnel testing, numerical simulation methods have been employed by researchers to investigate the aerodynamic characteristics of transmission conductors under combined wind and ice conditions. Sokolov et al. [11] used ANSYS FENSAP-ICE software to investigate bare and ice-coated conductors under unsteady wind fields, but did not conduct detailed calculations of ice accretion parameters. Regarding the influence of flow field characteristics on aerodynamic forces, Norberg et al. [12] demonstrated that both aerodynamic force coefficients and pressure coefficients in two-dimensional flow fields exhibit pronounced Reynolds number effects. Meanwhile, Gjelstrup et al. [13], Koss et al. [14], and Demartino et al. [15] have demonstrated that the aerodynamic coefficients of iced conductors correlate with two-dimensional flow characteristics and the Reynolds number. T. Ishihara et al. [16] developed a three-dimensional finite element model of iced conductors to investigate aerodynamic coefficients under varying ice thicknesses and bundle configurations. The model’s accuracy was experimentally validated, revealing that simulation results showed strong agreement with wind tunnel data when the spanwise length reached 10 times the conductor diameter.

Building upon research on the aerodynamic characteristics of ice-coated conductors, scholars worldwide have investigated galloping mechanisms and mitigation techniques for ice-coated transmission line conductors. Research on transmission line galloping primarily encompasses galloping modeling and dynamic response characteristics. Research on galloping models has undergone development from theoretical single-degree-of-freedom (SDOF) systems to multi-degree-of-freedom models using the finite element method [17], resulting in a well-established theoretical framework. For galloping response analysis of transmission lines, Chen et al. [18] investigated the displacement response of ice-coated conductors in turbulent stochastic wind fields using the probability density function. Their results demonstrated that the vibration amplitude under turbulent conditions is significantly greater than that under uniform flow conditions. Zhang et al. [19] and Alvise et al. [20] studied the impact of mountainous terrain wind conditions, ice thickness, and other factors on the ice accretion of transmission line conductors. Their studies revealed variation patterns of key parameters, including conductor vibration amplitude. Meng et al. [21–23] established a probabilistic damage assessment framework for transmission lines based on regional climatic characteristics. Their results confirm that combined wind-ice loads significantly increase galloping amplitudes. Critically, existing research on the galloping responses of transmission lines frequently fails to account for the effects of torsional stiffness. This omission leads to unreliable assessments of transmission line galloping performance.

In summary, existing studies on galloping of transmission conductors under wind loads rarely consider the effects of spanwise length, and few investigations have examined how torsional stiffness influences galloping responses. This study employs a three-dimensional numerical model to investigate the influence of wake effects and the angle of wind attack on the aerodynamic characteristics of bundled conductors with varying spanwise lengths. Furthermore, numerical simulations were employed to analyze variations in galloping amplitude of transmission conductors under different spacer connection configurations. It proposes a method for calculating the galloping response of iced transmission conductors that takes torsional stiffness into account. This approach revealed the failure mechanism of ice-coated transmission conductors under wind loads.

## 2. Governing equations and numerical method

### 2.1. Flow equations

The incompressible flow in this study is governed by the Navier-Stokes equations. Within the Arbitrary Lagrangian-Eulerian (ALE) reference frame, these equations are expressed as follows [24]:


$$\frac{\partial u}{\partial t}+\nabla \cdot \left(\left(u-w\right)u\right)=-\frac{\text{1}}{\rho }\nabla p+\nu \nabla^{2}u$$ (1)

∇·u=0 (2)

In the equations, u denotes the Reynolds-averaged fluid velocity vector, w represents the mesh velocity, p is the time-averaged pressure, υ is the kinematic viscosity, and ρ is the fluid density.

### 2.2. Structure equation

Galloping of iced transmission conductors constitutes a typical problem of nonlinear geometric vibration. Its motion characteristics manifest as large deformations occurring in the transverse plane, while exhibiting small-strain, linear elastic tensile deformation along the axial direction. Based on the updated Lagrangian formulation, the equations of motion for iced conductors under stochastic wind fields were established by Liu et al [25].


$$m\ddot{y}+c\dot{y}+ky=F_{y}$$ (3)

Where m is cylinder mass, c is the damping coefficient, and k is the structural stiffness. The variables $\ddot{y}$, $\dot{y}$ and y represent the transverse acceleration, velocity, and displacement of cylinders, respectively. The fluid dynamic force $F_{y}$ acting on the cylinder in the cross-flow direction is dependent on the cylinder’s motion.

In wind-induced vibration analysis of flexible conductors, damping serves as the primary mechanism for dissipating structural energy. The Rayleigh damping model is adopted, wherein damping is decomposed into mass-proportional and stiffness-proportional components to facilitate analytical treatment, expressed as:


c=αm+βk (4)

Where are the mass α and stiffness β damping coefficients, which are derived from the total damping ratio of the system ξ and the first two modal frequencies ($w_{1}$, $w_{\text{2}}$). The total damping ratio ξ of the transmission line system is taken as 1% [26]. The formulas for calculating these coefficients are:


$$\alpha =\xi \frac{\text{2}w_{1}w_{\text{2}}}{w_{1}+w_{\text{2}}}$$ (5)

$$\beta =\zeta \frac{\text{2}}{w_{1}+w_{\text{2}}}$$ (6)

Both vertical and horizontal vibration frequencies obtained through numerical simulation measured 0.46 Hz. Based on the calculation formula, the mass damping coefficient was determined as 0.00289 and the stiffness damping coefficient as 0.00346.

In this study, we numerically solved the governing equations (Eq. 3) using the commercial CFD software ANSYS Fluent, which incorporates the Shear Stress Transport (SST) k–ω turbulence model. The SST k-ω turbulence model, which solves transport equations for turbulent kinetic energy (k) and specific dissipation rate (ω), has demonstrated broad applicability through empirical validation against experimental data for bluff body flows [24]. The numerical discretization scheme was configured as follows: the pressure-velocity coupling was solved using the SIMPLE algorithm, the transient terms were discretized with a second-order implicit scheme, and both the turbulent kinetic energy (k) and specific dissipation rate (ω) were discretized via a second-order upwind scheme. Upon achieving flow field convergence at each time step, the structural response in the fluid-structure interaction (FSI) system was computed using the Newmark-β time integration method implemented through a User-Defined Function (UDF) [27]. The Newmark-β algorithm defines a time-marching relationship between the current state variables (displacement $y_{n}$, velocity ${\dot{y}}_{n}$, and acceleration ${\ddot{y}}_{n}$) and their values at the next time step t+Δt, where Δt is the time step increment. Specifically, the formulas for instantaneous velocity and displacement are defined as [28]:


$$\begin{array}{c} {\dot{y}}_{n+1}={\dot{y}}_{n}+\left[(1-\gamma ){\ddot{y}}_{n}+\gamma {\ddot{y}}_{n+1}\right]\Delta t \end{array}$$ (7)

$$\begin{array}{c} y_{n+1}=y_{n}+{\dot{y}}_{n}\Delta t+\left[(\frac{\text{1}}{\text{2}}-\beta ){\ddot{y}}_{n}+\beta {\ddot{y}}_{n+1}\right]\Delta t^{2} \end{array}$$ (8)

In this formula, γ and β denote the influence coefficients for velocity and displacement at time $t_{n+1}$, respectively. Using Equations (7) and (8), we can determine the velocity and acceleration at time $t_{n+1}$.Consequently, the displacement at time $t_{n+1}$ is given by:


yn+1=F~yn+1/F~yn+1k~\nulldelimiterspacek~ (9)

In this formulation, ${\tilde{F}}_{y_{n+1}}$ and $\tilde{k}$ denote the equivalent force and equivalent stiffness, respectively. where a0=1/1(βΔt2)\nulldelimiterspace(βΔt2), a1=γ/γ(βΔt)\nulldelimiterspace(βΔt), a2=1/1(βΔt)\nulldelimiterspace(βΔt), a3=1/1(2β)\nulldelimiterspace(2β)−1, $a_{7}=\gamma \Delta t$, a5=(γ/γβ\nulldelimiterspaceβ−2)Δt/(γ/γβ\nulldelimiterspaceβ−2)Δt2\nulldelimiterspace2, $\begin{array}{c} a_{6}=\left(1-\gamma \right)\Delta t \end{array}$, $a_{7}=\gamma \Delta t$. Consequently, the equivalent force and equivalent stiffness are given by:


$$\tilde{k}=a_{0}m+a_{1}c+k$$ (10)

$${\tilde{F}}_{y_{n+1}}=F_{y_{n+1}}+m\left(a_{0}y_{n}+a_{2}{\dot{y}}_{n}+a_{3}{\ddot{y}}_{n}\right)+c\left(a_{1}y_{n}+a_{4}{\dot{y}}_{n}+a_{5}{\ddot{y}}_{n}\right)$$ (11)

Therefore, the velocity and displacement at time $t_{n+1}$ are expressed as:


$${\dot{y}}_{n+1}={\dot{y}}_{n}+a_{6}{\ddot{y}}_{n}+a_{7}{\ddot{y}}_{n+1}$$ (12)

$${\ddot{y}}_{n+1}=a_{0}\left(y_{n+1}-y_{n}\right)-a_{2}{\dot{y}}_{n}-a_{3}{\ddot{y}}_{n}$$ (13)

### 2.3. Computational domain and boundary conditions

**Fig 1:**
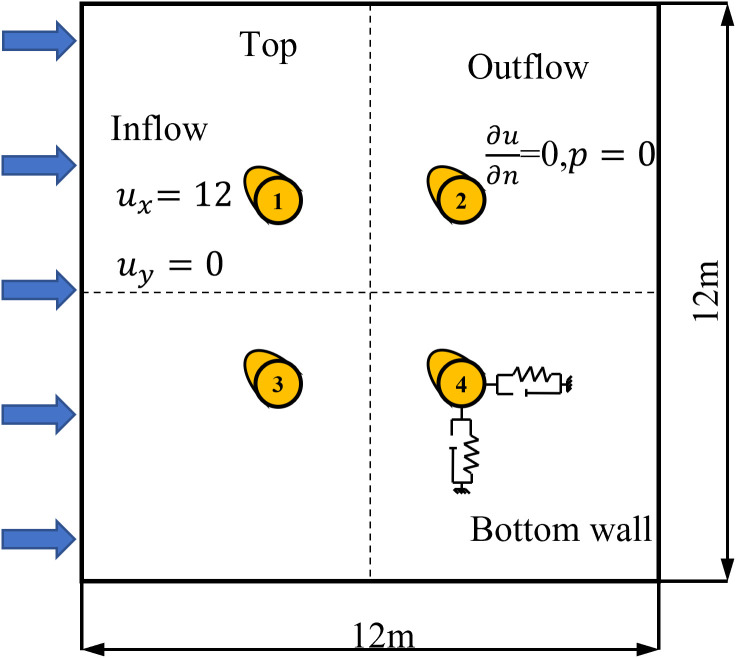
2D computational domain for flow around an ice-coated conductor.

To investigate the influence of spanwise length on the aerodynamic forces acting on the conductor and to compare the differences between two-dimensional and three-dimensional models, calculations were performed for the 2D model and for 3D models with spanwise lengths of 1 and 3 times the conductor diameter. The computational domain for the two-dimensional model is a square region. The specific configuration and boundary conditions are shown in Fig 1. The computational domain has a streamwise length and spanwise width both measuring 12 m, with D denoting the diameter of the cylindrical conductor. Detailed parameters of the conductor are provided in Table 1. In the bundled conductor configuration, the center-to-center spacing between adjacent sub-conductors is 0.45 m. Sub-conductor 1 is positioned 5.75 m downstream from the inflow boundary, while Sub-conductor 2 is located 5.75 m upstream from the outflow boundary. Navrose et al. [29] demonstrated that aerodynamic effects in the flow past a circular cylinder become negligible when the domain width is 20 D and the blockage ratio is 5%.

**Table 1:** Transmission Line Conductor Parameters.

Terms	Category	Elastic modulus (N/mm^2^)	Diameter (mm)	Cross section area (mm^2^)	Section ratio of steel to aluminum
Conductor	LGJ-400/50	73000	22.5	338.99	7.71

**Fig 2:**
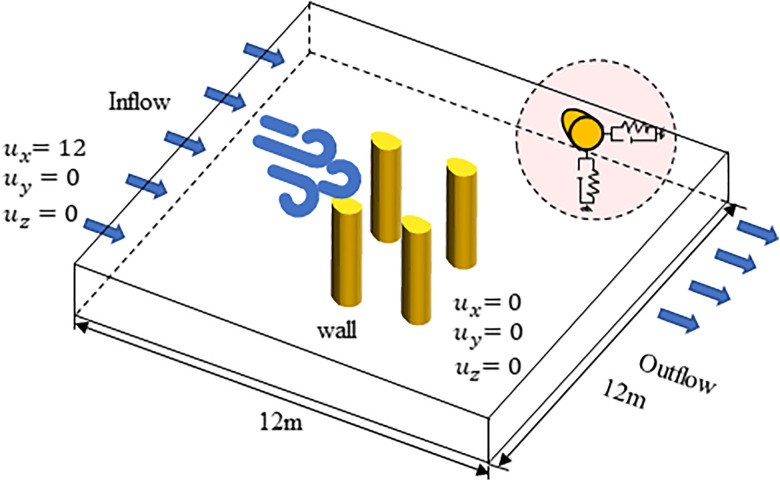
3D computational domain for flow around an ice-coated conductor.

The three-dimensional simulations use two distinct spanwise lengths of 3D and 1D, as schematically represented in Fig 2. Studies by Bruno [30] and Zhang et al. [31] demonstrate that the optimal spanwise domain length not only effectively captures three-dimensional flow characteristics but also maintains high computational efficiency. Yan et al. [32] employed similar boundary conditions in previous studies. The findings of E. Achenbach [33] and Hu et al. [34] further demonstrate that this computational domain effectively eliminates the influence of upstream and downstream far-field effects. In ANSYS Fluent, the inlet boundary condition was set to a velocity inlet with $u_{x}=12m/s,u_{y}=0$, and turbulence intensity I=5%*.* The outlet boundary condition was defined as a pressure outlet (∂u/∂u∂y\nulldelimiterspace∂y=0,υ=0). The ice-coated conductor surface was modeled as a stationary no-slip wall.

### 2.4. Research on grid division and time step convergence

**Fig 3:**
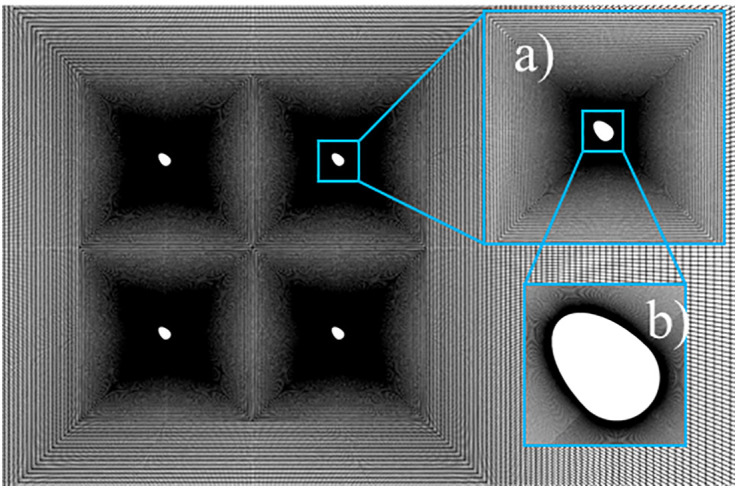
Computational domain grid (main figure). **(a)** Local grid of the ice-covered conductor; **(b)** Local grid of the ice-covered sub-conductor.

The computational domain mesh was generated using the structured mesh generation tool Automatic Mesh and the hexahedral mesh partitioning tool Hexahedral within the Ansys Workbench software. To accurately simulate flow near the model, a systematic mesh sensitivity analysis was conducted. Two mesh discretization methods with varying densities were employed to investigate the influence of the first-layer mesh height near the wall and the $y^{+}$ values on the aerodynamic characteristics of the split conductor. (1) Mesh 1: When the near-wall $y^{+}<1$, the local mesh near the iced conductor model was refined. The first-layer mesh height in the wall boundary layer was set to approximately 0.005 D, with a grid expansion factor of 1.2 applied to the encryption region to reduce computational costs. (2) Mesh 2: When the near-wall $1<y^{+}<5$, the first-layer mesh height near the wall was set to 0.01 D, and a grid expansion ratio of 1.2 was applied to the encryption region. For the 3D model, the x-y plane retained the same mesh configuration as the 2 D case. The spanwise lengths were set to 3D and 1 D in two distinct configurations, with the spanwise direction uniformly discretized into 6 cells in both cases. A grid independence study confirmed that the adopted mesh resolution has negligible effects on airflow and droplet dynamics, thereby satisfying the computational accuracy requirements. The computational domain and mesh configuration are illustrated in Fig 3.

This study adopts the classical aerodynamic theory of McComber et al. [35] to examine the relationship between ice thickness variations and aerodynamic coefficients. The crescent-shaped ice-covered cross-section is defined by the standard elliptic equation: $x^{2}/{\left(R+h\right)}^{2}+y^{2}/y^{2}=1$, (x≥0), with a 12 mm ice thickness serving as the baseline configuration. The geometry of the D-shaped ice accretion (commonly encountered in engineering applications) is represented by a parabolic equation: y=±D/D21−(x−h/x−hh\nulldelimiterspaceh)2\nulldelimiterspace21−(x−h/x−hh\nulldelimiterspaceh)2, (−h≤x<0).

Paidoussis et al. [36] performed a wind-tunnel experiment investigating the wake effects of cylindrical structures. Their results demonstrate that the presence of a downstream body significantly alters the vortex-induced vibration (VIV) process. Furthermore, Chen et al. [37] applied the Den Hartog galloping criterion to demonstrate that the aerodynamic coefficients of sub-conductors in bundle configurations ultimately govern their collective aerodynamic behavior. This result indicates that ice-bundled conductors exhibit significantly higher susceptibility to galloping than single ice-coated conductors. In summary, this study selected an ice-coated quad-bundled conductor as the research subject, with α = 45° adopted as the initial angle of attack.

Using the standard k-ω SST turbulence model, we analyzed different mesh configurations through comparative studies, with the primary computational cases summarized in Table 2. Prior to the transient analysis, a steady-state simulation of the ice-coated conductor was conducted using the RANS-based Realizable k-ω turbulence model. This simulation aimed to establish stabilized flow conditions as the initial state for transient computation, which accelerated the wake region’s progression into a fully developed phase. For the mesh convergence study, a constant temporal discretization scheme was employed to ensure time-step independence, with the transient simulations used a time step of Δt=0.0005s.

**Table 2:** Mesh configurations and computational cases.

Mesh	Spanwise length	Cell count	First layer grid	Near wall	Model
1Mesh-k		720000	0.003D	y+<1	K-ωSST
2Mesh-k		360000	0.005D	1 < y+<5	K-ωSST
1Mesh-k-1D	1D	2400000	0.005D	y+<1	K-ωSST
2Mesh-k-1D	1D	2160000	0.01D	1 < y+<5	K-ωSST
1Mesh-k-3D	3D	2865600	0.005D	y+<1	K-ωSST
2Mesh-k-3D	3D	717600	0.01D	1 < y+<5	K-ωSST

### 2.5. Mesh independence and model validation

To verify model convergence, a comparative study of flow around a circular cylinder was conducted at two Reynolds numbers: Re = 3,900 and Re = 5.9 × 10⁴. For the comparative group, the preset simulation methodology was employed, and the comparison of the simulation results with the experimental data is presented in Table 3. The drag coefficient $C_{D}$, lift coefficient $C_{L}$, and Strouhal number St are defined as follows:

**Table 3:** The numerical simulation results are compared with the literature results.

Working condition	St	C_D,mean_	C_p_	Working condition	St	C_D,mean_	C_l,mean_
F.TREMBLAY [38]	0.220	1.03	−0.92	ÁLVAREZ [39]	0.142	1.031	−0.026
1Mesh-k	0.20(6.9%)	0.94(8.8%)	−0.87(5.5%)	1Mesh-k	0.149(5.5%)	1.103(7%)	−0.0246(5.5%)
2Mesh-k	0.201(8.5%)	0.92(10.6%)	−0.86(5.5%)	2Mesh-k	0.154(8.5%)	1.113(8%)	−0.0246(5.5%)
1Mesh-k-1D	0.22(0.4%)	1.01(2.2%)	−0.92(1.0%)	1Mesh-k-1D	0.1424(0.3%)	1.054(2.2%)	−0.0258(0.9%)
2Mesh-k-1D	0.216(1.7%)	0.99(3.6%)	−0.89(3.5%)	2Mesh-k-1D	0.144(1.3%)	1.054(2.2%)	−0.0252(3%)
1Mesh-k-3D	0.219(0.4%)	1.008(2.1%)	−0.91(0.9%)	1Mesh-k-3D	0.1424(0.3%)	1.054 (2.2%)	−0.0258(0.8%)
2Mesh-k-3D	0.218(0.6%)	1.004(2.5%)	−0.895(2.7%)	2Mesh-k-3D	0.1424(0.3%)	1.053(2.1%)	−0.0257(1%)


$$C_{D}=\frac{F_{D}}{\frac{\text{1}}{\text{2}}\rho U^{2}LD}$$ (14)

$$C_{L}=\frac{F_{L}}{\frac{\text{1}}{\text{2}}\rho U^{2}LD}$$ (15)

$$C_{p,i}=\frac{P_{i}}{\frac{\text{1}}{\text{2}}\rho U^{2}}$$ (16)

$$St=\frac{fD}{U_{0}}$$ (17)

Where $F_{D}$ and $F_{L}$ are the drag and lift forces acting on the cylinder, ρ is the fluid density, U is the free-stream velocity, L is the length of the ice-coated conductor, and D is the diameter of the bare conductor. where f represents the vortex shedding frequency, which can be determined from spectral analysis of the lift coefficient fluctuations. Table 3 presents the average drag coefficient and the wind pressure coefficient. As shown in Table 3, the absolute mean values and standard deviations of lift and drag coefficients obtained from the Mesh-k-1D simulation results differ by less than 11% from experimental and simulated results reported in previous literature. The St value exhibits a discrepancy of less than 3% compared to simulation results reported in previous literature. This validates the accuracy of the numerical methodology. Furthermore, simulation results remained virtually unchanged upon further mesh refinement. By systematically balancing computational accuracy with resource efficiency, we adopted the 1Mesh-k-1D configuration for all icing conductor simulations in subsequent studies.

**Fig 4:**
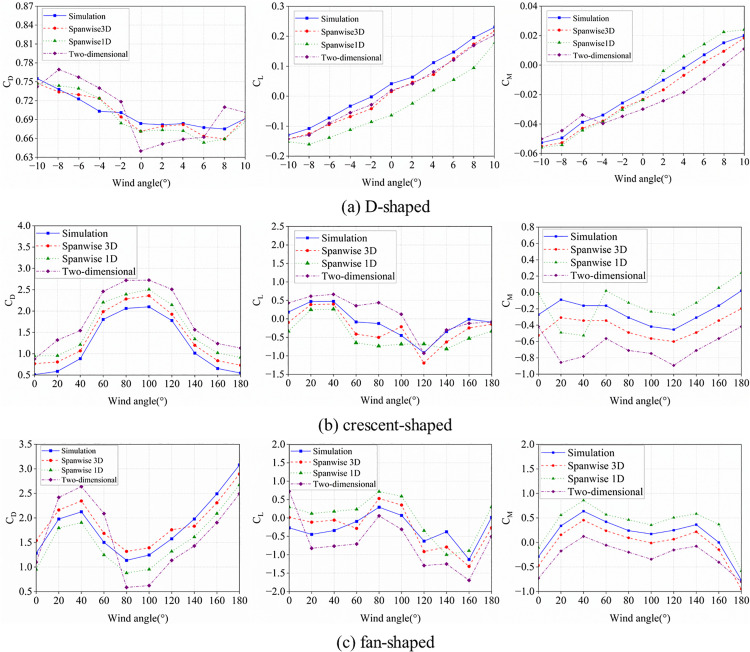
Three-component aerodynamic coefficients for iced conductor.

**Fig 5:**
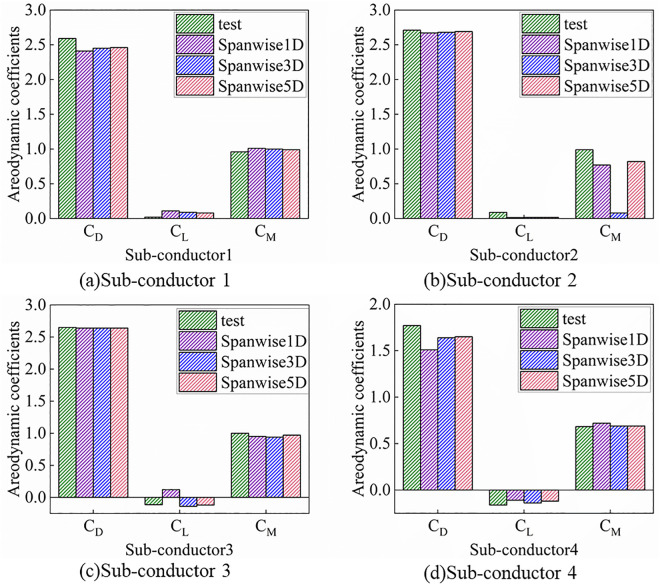
Aerodynamic coefficients of the quad bundle conductor with crescent-shaped ice (wind speed: 10 m/s; ice thickness: 20 mm).

Prior to investigating the aerodynamic characteristics of ice-accreted conductors, numerical simulations for D-shaped, crescent-shaped, and fan-shaped ice accretions were conducted and compared against wind tunnel test data [40, 41] to validate the Computational Fluid Dynamics (CFD) methodology. As shown in Fig 4, the CFD technique inherently exhibits certain errors in resolving small-scale vortices. However, in terms of overall variation patterns, the numerical simulation results demonstrate good agreement with the wind tunnel experimental results. The computational results with Mesh 1 are superior to those of Mesh 2. When the spanwise length is set to one time the characteristic length, satisfactory computational accuracy is achieved. Increasing the spanwise length to three times the characteristic length yields no significant improvement in computational precision. Additionally, Yang et al. [42] conducted a convergence analysis of the numerical method and validation for vortex-induced vibrations (VIV) of a circular cylinder. The results demonstrate that the proposed simulation method accurately captures the overall trends and variation patterns of aerodynamic characteristics in ice-covered conductors observed in both experimental tests and numerical simulations, confirming its suitability for numerical analysis of conductor aerodynamic behavior. To validate the applicability of this model in aerodynamic analysis of bundled conductors, we and Hu et al [43,44]. conducted aerodynamic analysis on the same ice-covered quad-bundled conductor and compared the numerical results, as shown in Fig 5. The numerical results demonstrate close agreement with experimental data, thus validating the accuracy of the three-dimensional numerical model.

## 3. Numerical simulation of aerodynamic characteristics of iced bundled conductors

This section analyzes the effects of various physical parameters of iced bundled conductors on their aerodynamic characteristics. The analysis primarily encompasses the aerodynamic response of iced bundled conductors under varying parameters including wake effects, ice morphology, ice thickness, angle of attack, and wind speed. To better illustrate the impact of mesh parameters on aerodynamic numerical simulation results, the model parameter 1 Mesh-k-1 D was selected.

### 3.1. Aerodynamic impact of wake effects on iced bundled conductors

**Fig 6:**
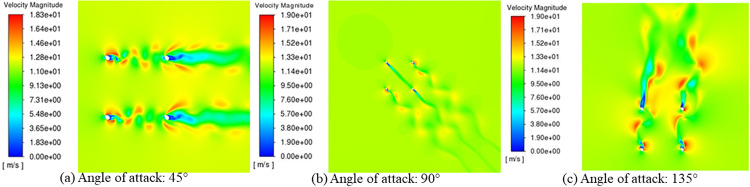
Velocity contours of air flow around the iced-quad bundle conductor at three angles of attack (t_i_=12 mm, U=12 m/s).

When wind loads act on iced bundled conductors, periodic vortex shedding occurs in the near wake, which triggers a structural vibrational response due to wake effects. Fig 6 illustrates the velocity contours of the flow field around iced bundled conductors under a fixed wind speed of 12 m/s and ice thickness of 12 mm, with varying angles of attack. As illustrated in Fig 6, as the angle of attack is 45°, the wake effects from sub-conductors 2 and 4 significantly affect on sub-conductors 1 and 3, leading to pronounced velocity differences between their windward and leeward sides. It is observed that the flows around sub-conductors 4 are apparently influenced by the wake of sub-conductors 1, respectively, as the angle of attack is 90°, while negligible interactions are observed among the remaining sub-conductors. As the angle of attack is 135°, sub-conductors 1 and 2 are influenced by the wake effects from sub-conductors 3 and 4. As the angle of attack is 45° and 135°, sub-conductors within the bundle exhibit the most pronounced wake effects. The leeward sub-conductor experiences aerodynamic sheltering from the windward sub-conductor, reducing flow velocity around the leeward sub-conductor and consequently modifying its aerodynamic coefficients. These phenomena serve as preliminary indicators for assessing wind loads and galloping behavior of bundled conductors under varying angle of attacks.

As revealed by the analysis of wake effect influence, when designing sub-conductor systems to ensure stability and fatigue resistance, rigorous evaluation of wake-induced impacts must be prioritized. Full consideration should be given to the degradation of vibrational stability and fatigue life caused by wake effects.

### 3.2. Investigation into aerodynamic coefficient patterns of ice-covered bundled conductors

#### 3.2.1. Variation characteristics of aerodynamic coefficients with angle of attack.

Fig 6 depicts the variations in aerodynamic coefficients with angles of attack for crescent-shaped ice-accreted quadruple bundled conductors under a wind speed of 12 m/s and an ice thickness of 12 mm.

**Fig 7:**
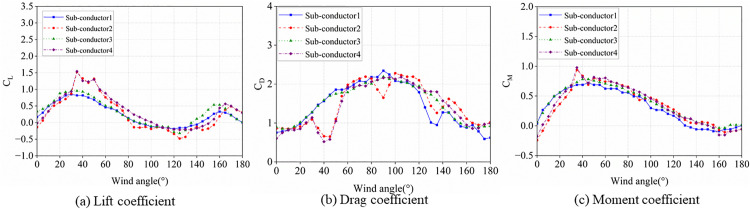
Aerodynamic coefficients of the iced conductor varying with an angle of attack. (t_i_ = 12 mm, U = 12 m/s).

As illustrated in Fig 7(a), the lift coefficient curve displays an S-shaped fluctuation. Owing to the symmetry of the ice-accreted cross-section on the conductor model, the lift coefficient approaches zero at angles of attack of 0° and 180°, consequently generating no lateral lift forces. Within a defined range of angles of attack, the lift coefficient increases with the angle until reaching its peak value. As the angle of attack further increases, the lift coefficient transitions from positive to negative values, indicating a reversal of the aerodynamic lift force direction to vertically downward. As shown in Fig 7(b), sub-conductors 1 and 3 exhibit half-wave sinusoidal drag coefficient curves, small at both ends and large in the middle, whereas those of sub-conductors 2 and 4 display pronounced M-shaped distributions featuring twin peaks separated by a trough. This phenomenon is attributed to the complete shielding of the quad-bundled conductors at a 90° angle of attack, where the wake effect alters the drag coefficient, resulting in a reduction of the coefficient for the leeward conductor. As demonstrated in Fig 7(c), the torque coefficient approaches zero at angle of attack of 0° and 180°, a direct consequence of the symmetric ice accretion on the conductor model. The torque coefficient is largely unaffected by wake effects, and the variation patterns of torque coefficients across sub-conductors with wind attack angles are generally consistent. The aforementioned studies demonstrate that the wake effects of bundled conductors exert a lesser influence on the lift coefficient compared to their impact on the drag coefficient.

#### 3.2.2. Variation patterns of aerodynamic coefficients with ice accretion shapes.

**Fig 8:**
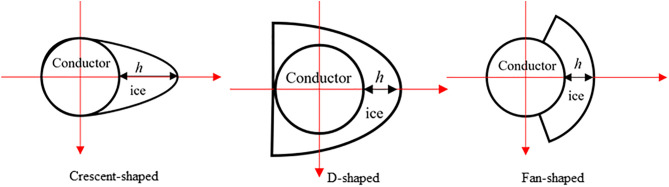
Different icing cross-section shapes.

During ice accretion on transmission conductors, variations in meteorological conditions and span lengths lead to differential torsional rigidity, resulting in diverse ice accretion morphologies, such as elliptical, circular, and D-shaped configurations. For conductors with elevate stiffness, elongated ice accretion or other irregular configurations develop predominantly on the windward side, while little to no ice accumulation occurs on the leeward side. Common forms include crescent or fan shapes. To investigate the variation patterns of aerodynamic coefficients with ice shapes in Xinjiang, three representative ice shapes (crescent-shaped, D-shaped, and fan-shaped) with uniform 12 mm thickness were selected based on the 50-year recurrence interval ice zone distribution map of the region, as illustrated in Fig 8.

**Fig 9:**
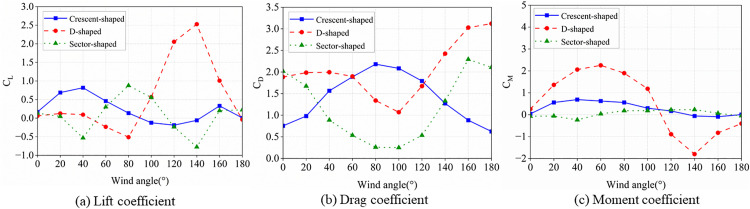
Relationship between aerodynamic force coefficients and ice accretion shapes (Sub-conductor 1, U = 12 m/s).

Fig 9 demonstrates the variation of aerodynamic coefficients for Sub-conductor 1 with ice accretion shapes under 12 m/s wind velocity. As evident from Fig 9, the magnitudes of aerodynamic coefficients for bundled conductors with equivalent ice thickness-to-diameter exhibit monotonic escalation with increasing angles of attack within specific angular ranges. This aerodynamic behavior is predominantly governed by the enhanced windward projected area of D-shaped ice accretions compared to crescent and fan-type shapes. For crescent-shaped ice accretion, within specific angle of attack ranges, the forward shift of flow separation points induces a significant increase in windward surface area. This phenomenon enhances the drag force acting on the conductor surface, consequently leading to a progressive rise in aerodynamic drag coefficient. The magnitude of aerodynamic force coefficients is governed by the windward surface area of the conductor. Consequently, conductors with different ice accretion geometries exhibit distinct aerodynamic characteristics.

#### 3.2.3. Variation patterns of aerodynamic coefficients with ice thickness.

**Fig 10:**
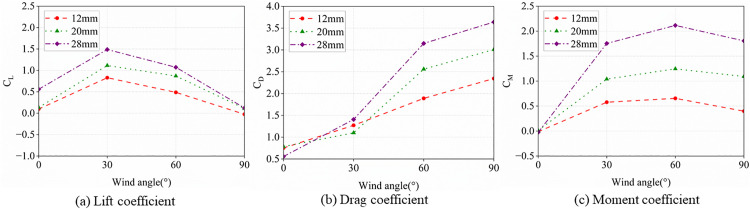
Variation of aerodynamic coefficients with ice thickness (Sub-conductor 1, U = 12 m/s).

Fig 10 presents the variation of aerodynamic force coefficients with ice thickness for conductors exhibiting crescent-shaped ice accretion. Fig 10(a) and (b) demonstrate that both lift and drag coefficients exhibit nearly identical variation patterns across different ice thickness conditions; Within 0° ~ 90° angles of attack, both the lift and drag coefficients increase with ice thickness. Under these conditions, ice thickness significantly influences the aerodynamic lift and drag forces on the conductor, thereby affecting the galloping behavior of bundled conductors. As demonstrated in Fig 10(c), the torque coefficient approaches zero at 0° angle of attack due to the symmetry of the ice accretion. Under thin ice accretion conditions, the conductor cross-section remains nearly circular, resulting in a smaller torque coefficient with minimal variation across angles of attack. However, as ice thickness increases, the torque coefficient exhibits a pronounced upward trend.

#### 3.2.4. Variation patterns of aerodynamic coefficients with wind speed.

**Fig 11:**
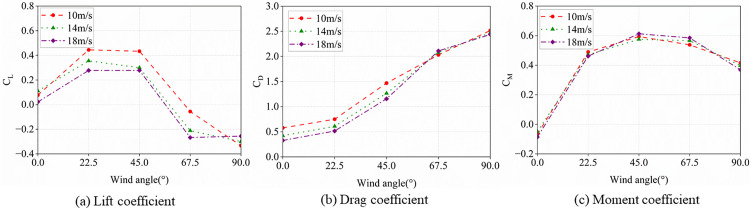
Variation of aerodynamic coefficients with wind speed.

As shown in Fig 11, which depicts the aerodynamic coefficients of a crescent-shaped iced conductor (12 mm thickness) versus wind attack angle under various wind conditions, the overall variation patterns remain largely consistent across different wind speeds. This phenomenon is attributed to the fact that within the wind speed range of 10 m/s to 18 m/s, the conductor Reynolds number remains in the subcritical regime, where the flow field characteristics governing aerodynamic coefficient variations are insensitive to Reynolds number changes. Although the curve morphology of dimensionless aerodynamic coefficients exhibits negligible variation, the actual aerodynamic forces acting on the conductor vary quadratically with wind speed according to the aerodynamic calculation formulas (Equations 14–17). Consequently, increased wind speed substantially increases the aerodynamic load, which leads to larger conductor galloping amplitudes. Notably, galloping amplitudes of ice-accreted conductors depend not only on wind speed but typically peak within a specific critical wind speed range.

In summary, the flow-around characteristics of ice-coated bundled conductors are governed by the interaction of multiple factors, including wake effects, wind attack angle, ice accretion morphology, ice thickness, and wind speed. These findings provide a theoretical reference for galloping numerical simulation analysis of transmission lines and galloping prevention/early-warning strategies.

## 4. Galloping analysis of ice-coated bundle conductors

To evaluate the effectiveness of numerically derived aerodynamic coefficients in the analysis of ice-induced galloping of transmission lines. Utilizing numerically simulated aerodynamic coefficients, this study analyzes the nonlinear galloping behavior of actual ice-coated bundle conductors in Xinjiang’s power grid based on elastic catenary theory.

### 4.1. Numerical simulation method of galloping

Based on the updated Lagrange formulation, the galloping equation of ice-coated multi-bundled conductors can be expressed by Equation (3). The mass matrix for an ice-coated bundle-conductor element is defined as:


$$m_{e}=\int_{0}^{l_{e}}N^{T}\mu Nds$$ (18)

$$\mu =\int_{A_{T}}\rho R^{T}RdA$$ (19)

$$R=\left[\begin{array}{cc} {}*20c1 & -z\\ 0 & y \end{array}\right]$$ (20)

where ρ denotes the volumetric mass density of the ice-coated conductor, $A_{T}$ represents its cross-sectional area, and $l_{e}$ is the element length. The stiffness matrix $k_{e}$ for an ice-coated transmission line conductor element is defined as:


$$k_{e}=k_{e}^{l}+k_{e}^{n}+k_{e}^{ice}$$ (21)

Within the local coordinate system, the stiffness matrix of the ice-coated transmission line element comprises three components: $k_{e}^{l}$ denotes the conductor’s elastic stiffness matrix, $k_{e}^{n}$ its geometric stiffness matrix, and $k_{e}^{ice}$ ice the additional ice-related stiffness component.


$$k_{e}^{l}=\int_{0}^{l_{e}}AB^{T}DBds=\int_{0}^{l_{e}}B^{T}\left[\begin{array}{cc} {}*20cAE & B_{T}\\ B_{T} & GJ \end{array}\right]Bds$$ (22)

$$B=\left[\;\;\;\begin{array}{cc} {}*20cB_{1} & B_{2} \end{array}\;\;\;\right]$$ (23)

$$B_{k}=\left[\begin{array}{cc} {}*20c\frac{\partial X^{*}}{\partial S}\frac{\partial N_{k}}{\partial S} & \text{0}\\ \text{0} & \frac{\partial N_{k}}{\partial S} \end{array}\right]$$ (24)

Where E is the elastic modulus, G is the shear modulus, $X^{*}$ denotes the coordinate of an arbitrary point on the element in the global coordinate system, and $N_{k}$ represents the shape function [17]. Where $GJ_{\min}=n\tau +R^{2}T$ and $GJ_{\max}=n\tau +R^{2}T+\frac{16r^{2}EA}{3L^{2}}{y_{0}}^{2}$ represents the additional stiffness induced by the sub-conductor tension difference due to spacer damper constraints. The detailed derivation process can be found in the literature.


$$k_{e}^{n}=\int_{\text{\textbackslash{}!}\;\;0}^{l_{e}}O^{T}SOds$$ (25)

$$O=\left[\;\;\;\begin{array}{cc} {}*20c\frac{\partial N_{1}}{\partial S}I & \frac{\partial N_{2}}{\partial S}I \end{array}\;\;\;\right],\;I=\left[\begin{array}{cc} {}*20c1 & 0\\ 0 & 0 \end{array}\right]$$ (26)

where O the derivation of the matrix can be found in Reference [17]; S denotes the axial force.


$$k_{e}^{ice}=-g\int_{0}^{l_{e}}N^{T}\left[\begin{array}{cc} {}*20c0 & 0\\ 0 & S_{Z} \end{array}\right]Nds$$ (27)

$$S_{z}=\sum_{i=1}^{2}N_{k}S_{zi},S_{zi}=\int_{A_{T}}\rho dA$$ (28)

where g is the gravitational acceleration. The aerodynamic force vector $F_{y}$ for galloping of an ice-coated bundled conductor element is expressed as:


$$F_{y}={\left[\;\;\;\begin{array}{cc} {}*20cF_{yi}^{1} & F_{yi}^{2} \end{array}\;\;\right]}^{T}$$ (29)

$$F_{yi}^{j}=\left[\;\;\;\begin{array}{cccc} {}*20c0 & F_{Li}^{j}\left(\alpha \right) & F_{Di}^{j}\left(\alpha \right) & M_{Mi}^{j}\left(\alpha \right) \end{array}\;\;\;\right]$$ (30)

Where $F_{yi}^{j}$ denotes the galloping aerodynamic force vector at the j node of the i sub-conductor unit; $F_{Li}^{j}$, $F_{Di}^{j}$ and $M_{Mi}^{j}$ represent the lift force, drag force, and aerodynamic moment at the j node of the i sub-conductor unit, respectively; α is the angle of attack on ice-coated conductors.

### 4.2. Finite-element model of iced-quad bundle conductor lines

**Fig 12:**
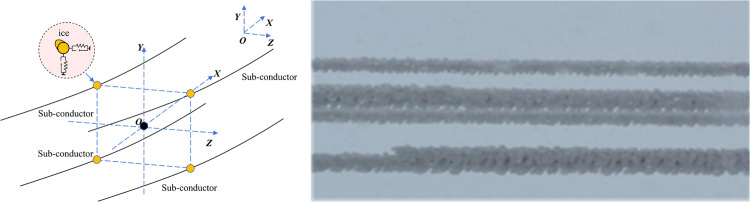
Schematic of Ice-Covered Multi-Bundled Transmission Line Conductor.

To better elucidate the impact of combined wind-ice loading on the performance of overhead transmission lines in Xinjiang, this study selects a 500 kV ice-coated quad-bundled conductor from the Xinjiang power grid as the research subject, as shown in Fig 12. This design has been extensively adopted across multiple regions in China, facilitating the identification of similarly designed power lines in Xinjiang. However, transmission lines traversing the mountain pass section in Dabancheng are susceptible to galloping phenomena under specific microclimatic conditions. Consequently, targeted structural modifications were implemented on this transmission line during subsequent operational phases. Comparative analysis of engineering design drawings from different regions revealed that while specifications of certain tower components exhibit regional variations, the conductor and insulator models remain identical. Table 1 details the conductor parameters.

An ice-covered bundled conductor finite element model was developed using transmission line design parameters from Dabancheng, Xinjiang. The model simulates a single-span transmission line configuration with a 300 m span length and a 92 KN mid-span conductor tension; The adjacent sub-conductor spacing is set at 450 mm. The conductors are modeled using cable elements. To constrain the relative motion between the nodes of the sub-conductors, spacers are simulated using rigid elements connecting the nodes, with their specific configurations detailed in Table 4. Furthermore, to ensure accuracy in simulating the initial catenary configuration of the transmission line, iterative calculations and repeated adjustments were performed on the model parameters, until the calculated sag under self-weight matched the design sag [45].

**Table 4:** Configuration Details of Spacers.

Conditions	1	2	3	4
Distance from Right End/m	240	180	120	60

#### 4.2.1. Model validation.

**Fig 13:**
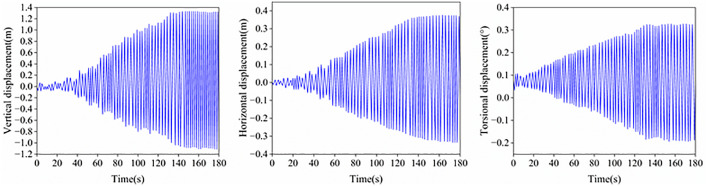
Time histories of displacements at the midspan of conductors.

Reference [38] analyzed the galloping response of this cross-span transmission conductor. To validate the accuracy of the galloping response calculation method proposed in this study, the identical computational scenario from Reference [39] was employed for dynamic time-history analysis, specifically with an initial angle of attack of 180°, wind speed of 6 m/s, initial sag of 1%, while both the elastic modulus E and cross-sectional area A of the bare conductor were assigned their mean values. Fig 13 illustrates the time histories of vertical, lateral, and torsional displacements at the conductor mid-span. Following the onset of periodic galloping, the vibration amplitudes of vertical, lateral, and torsional displacement curves at the mid-span position are comparatively analyzed against computational results from the Reference [39] model in Table 5. With all computational errors remaining below 5%, the accuracy of the galloping calculation method proposed in this study is demonstrated.

**Table 5:** Configuration Details of Spacers.

Amplitude	Reference [44]	This paper	Error (%)
Vertical displacement(m)	1.13	1.147	1.5
Horizontal displacement(m)	0.38	0.037	2.63
Torsional displacement/º	0.325	0.326	3.1

### 4.3. Galloping of ice-coated bundled conductors

#### 4.3.1. Effect of conductor stiffness.

**Fig 14:**
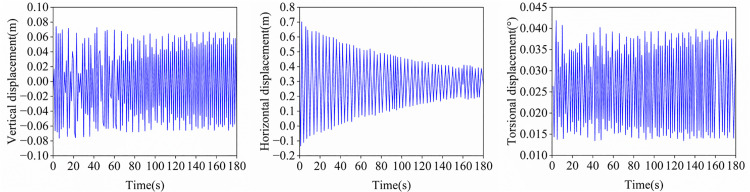
Simplified model mid-span displacement time history.

Using the finite element software ANSYS, an initial model of an equivalent single conductor devoid of initial stress and sag was established. Following the specification of structural and material parameters for the conductor, initial tension and gravitational loads were applied, enabling the determination of its static equilibrium configuration through form-finding analysis. Building upon this foundation, torsional stiffness $GJ_{\min}$ is incorporated into the conductor model. The simplified model requires the following assumptions:(a) Only full-span vibration of conductors is considered;(b) Sag-to-span ratio < 1:8;(c) Conductor torsion angle is minimal;(d) Rigid spacers at nodes maintain coplanar configuration;(e) Normal vector of spacer plane is tangent to the centroidal axis;(f) Synchronous longitudinal motion of sub-conductors;(g) Identical rotation angles between sub-conductors and centroidal axis;(h) Longitudinal inertial forces and damping forces of conductors are neglected. As illustrated in Fig 14, the conductor’s galloping amplitude remains relatively small during the 1–180 s period, with vertical displacement of approximately 0.07 m, horizontal displacement of ~0.7 m, and torsional amplitude of ~0.043°. Studies by Ge et al. [46] and Hisato et al. [47] reveal that conductor galloping primarily originates from: (1) modal coupling effects induced when torsional and vertical natural frequencies approach each other, and (2) coupled interactions between aerodynamic stiffness and damping. However, the simplified model adopted in this study neglects aerodynamic coupling effects and relative motion between sub-conductors. Consequently, it is restricted to simulating bundled conductors undergoing minor torsional deformation.

**Fig 15:**
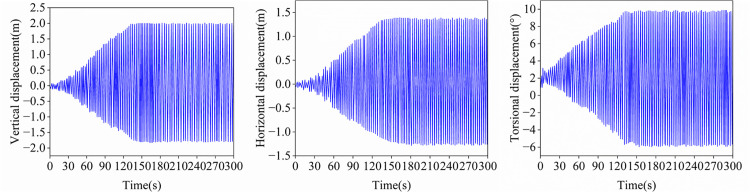
Time histories of displacements at the midspan of sub-conductor 1.

Using the finite element software ANSYS, an initial model of the quadruple-bundled conductor was established in the absence of initial stress and sag. Following the same procedure as for the simplified model, the vertical displacement $y_{0}$ of the conductor was obtained. Building upon this foundation, the galloping response of the bundled conductor was analyzed, accounting for the torsional stiffness effect arising from tension differences among sub-conductors induced by spacer damper constraints. It is demonstrated that a wind attack angle of 30° induces critical galloping in Sub-conductor No. 1, as evidenced in Fig 15. The initial low-amplitude oscillation evolves into a steady-state galloping response, characterized by vertical displacement, horizontal displacement, and torsional amplitude of 1.9 m, 1.38 m, and 9.8°, respectively. Comparative analysis demonstrates that the stiffness of bundled conductors constitutes the governing parameter triggering galloping oscillations. Specifically, the rigid constraints imposed by spacer dampers substantially suppress torsional amplitude development while amplifying horizontal displacements.

This study defines two critical damping parameters: the inherent damping ratio of transmission conductors, which represents their intrinsic structural energy dissipation capacity, determined through calculation using Equations (5)-(6), and the equivalent damping ratio of spacer dampers. Full-scale tests by Yan et al. [48] demonstrate that galloping suppression effectiveness achieves the optimum when the damping ratio is maintained within the 0.2–0.3 range. For the scenario with a wind speed of 12 m/s and ice thickness of 10 mm, an analysis of the influence of the spacer damper’s damping ratio was conducted, considering structural stiffness requirements. Therefore, galloping response analysis was performed on a quad-bundled, ice-covered conductor equipped with spacer dampers, with the damping ratio varied from 0.1 to 0.3. Through computational simulations with three damping ratios, the influence of damping ratio on conductor galloping amplitude was systematically examined. The displacement amplitudes of conductor galloping under varying damping ratios are detailed in Table 6.

**Table 6:** Effect of Damping Ratio on Galloping Amplitude.

Damping ratio	Galloping amplitude/m	
	Horizontal direction	Vertical direction
0.1	1.38	1.97
0.2	1.204	1.732
0.3	0.954	1.506

As indicated in Table 6, when the damping ratio is 0.3, the vertical galloping amplitude of the conductor decreases to 1.506 m and the horizontal amplitude to 0.954 m, demonstrating optimal galloping suppression performance of the system. Analysis demonstrates that increasing the damping ratio effectively improves the energy dissipation capacity in quad-bundled conductor systems, substantially reducing galloping amplitudes. In summary, the deployment of spacer proves to be an effective approach for suppressing galloping in ice-covered quad-bundled conductors. To achieve optimal galloping suppression performance, follow-up studies should conduct combinatorial optimization analysis of critical design parameters for the dampers.

#### 4.3.2. Effects of wind angle of attack.

**Fig 16:**
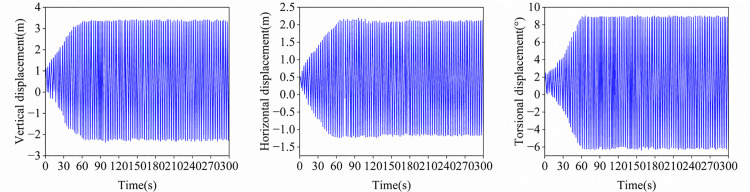
Time histories of displacements at the midspan of sub-conductor 1(α  = 45°).

The angle of attack constitutes the dominant governing parameter for galloping response in transmission tower-line systems. Research indicates that as the number of sub-conductors in a bundled conductor increases, the aerodynamic interference effects between them are significantly enhanced under various wind angles of attack. Therefore, to investigate the influence of wind angle of attack on conductor galloping, this section systematically analyzes the galloping response under identical line and meteorological parameters. Figs 16 and 18 present the galloping response time history of Sub-conductor 1 at two representative wind angles of attack (45° and 135°) at a wind speed of 12 m/s.

**Fig 17:**
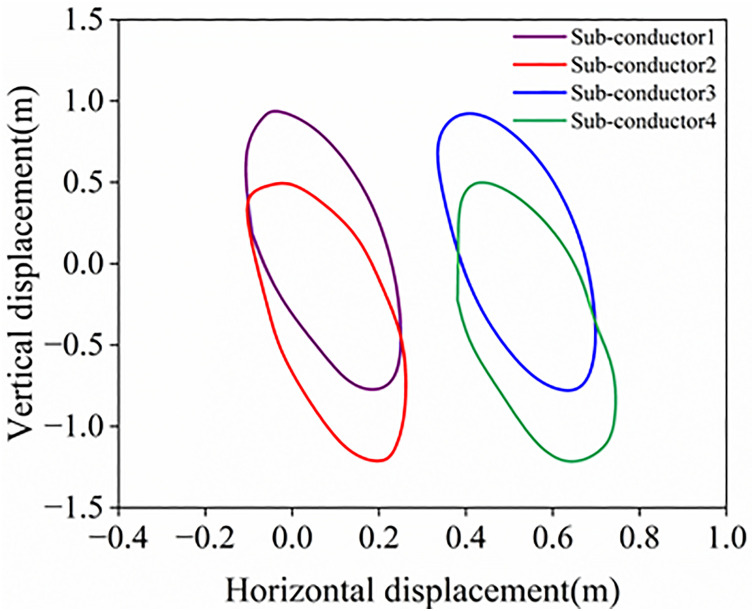
Galloping traces at the midspan of the conductor line.

When the wind angle of attack is 45°, Sub-conductor 1 initially exhibits small-amplitude vertical vibration centered on its equilibrium position. The aerodynamic negative damping effect leads to continuous accumulation of mechanical energy in the tower-line system, causing the system to enter a steady-state galloping stage 60 seconds after vibration initiation. The steady-state displacements at the mid-span node are approximately 3.38 m (vertical) and 2.18 m (horizontal), while the torsional amplitude ranges from −6.7° to 9°. It is noteworthy that the aforementioned displacements and torsional amplitude significantly exceed the corresponding values of a twin-bundle conductor under identical conditions. At the mid-span node, the ratio of vertical to horizontal displacements reaches 1.55. As shown in Fig 17, all ice-coated sub-conductors exhibit distinct elliptical trajectories, indicating that their galloping motion is dominated by the vertical component of wind velocity. This phenomenon conforms to the Den Hartog criterion for aerodynamic instability; it reveals that the primary cause of galloping onset is aerodynamic negative damping.

**Fig 18:**
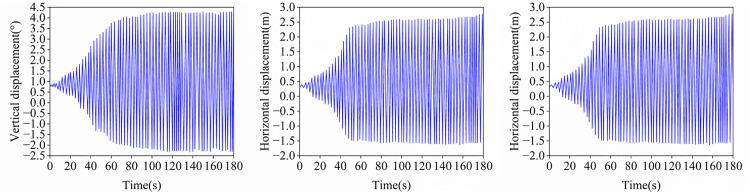
Time histories of displacements at the midspan of sub-conductor 1(α = 135°).

To evaluate the galloping characteristics of the sub-conductor under wake effects from adjacent conductors, the galloping response of sub-conductor 1 at a 135° angle of attack and 12 m/s wind speed was analyzed, as shown in Fig 18. At the mid-span node of Sub-conductor 1 under steady galloping conditions, the vertical displacement amplitude ranges from –2.33 m to 4.30 m, the horizontal displacement amplitude ranges from –1.64 m to 2.78 m, and the torsional amplitude ranges from –4.10° to 9.83°. Compared to the 45° angle of attack, the horizontal and torsional amplitudes are reduced at 135°, whereas the galloping characteristics remain qualitatively similar. The elliptical trajectory, predominantly characterized by vertical displacement, indicates that this galloping motion constitutes nonlinear vibration induced by vertical–horizontal coupling. Compared to twin-bundle conductors, this sub-conductor exhibits significantly greater galloping amplitudes in all degrees of freedom. Crucially, spacer dampers effectively suppress the torsional rotation of ice-coated sub-conductors, thereby enhancing the overall torsional stiffness of the bundled-conductor system. These results demonstrate the critical role of spacer dampers in suppressing galloping. Consequently, optimizing both the damper-conductor interface and mechanical properties provides a crucial approach to enhance galloping suppression effectiveness in transmission line field operation.

#### 4.3.3. Effect of spacer damper connection configuration.

As a critical component in bundled-conductor transmission systems, the spacer damper serves to maintain sub-conductor spacing, prevent collisions, and suppress wind-induced vibrations such as conductor oscillation, galloping, and aeolian vibration. However, vibrations induced by electrodynamic forces between sub-conductors and by wake-induced vibrations can cause mechanical failures in spacer dampers, compromising the structural stability of transmission lines. Consequently, comprehensive analysis of mechanical behavior at the conductor-spacer damper interface is critical to ensure structural reliability in power transmission systems.

**Fig 19:**
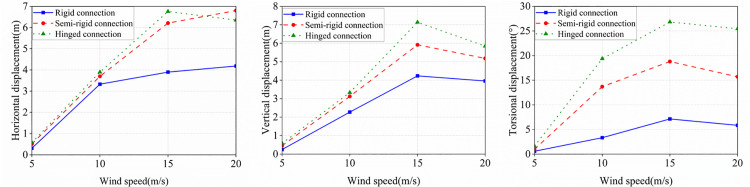
Galloping amplitude of a spacer under different connection modes.

As illustrated in Fig 19, conductor-spacer damper attachments primarily comprise three configurations: hinged joints, semi-rigid connections, and rigid clamps. Research demonstrates that spacer damper installation effectively mitigates torsional deformation in bundled conductor systems, while the joint constraint stiffness determined by their attachment configuration governs the global torsional natural frequency of the system. As shown in Table 7, such variations in the system torsional frequency significantly influence the galloping stability of ice-accreted conductors under wind excitation. Investigations into galloping amplitude-wind speed relationships reveal two characteristic regimes across attachment configurations: within the 5–15 m/s range, galloping amplitude exhibits monotonic increase, while between 15–20 m/s, it transitions to a monotonic decrease. Crucially, joint constraint stiffness exerts a pronounced modulating effect on galloping amplitude. Rigid connections exhibit the maximum galloping amplitude across all vibration directions; conversely, pinned connections demonstrate the minimum amplitude, while semi-rigid connections show intermediate galloping responses between these two extremes.

**Table 7:** Natural Frequencies of Bundled Conductors.

Connection modes	Direction	Modal shape	Frequency(Hz)
Rigid connection	Torsion	peak	0.9
	Horizontal		0.8
	Vertical		0.83
Semi-rigid connection	Torsion	peak	0.7
	Horizontal		0.62
	Vertical		0.68
Hinged connection	Torsion	peak	0.4
	Horizontal		0.32
	Vertical		0.33

In summary, a significant positive correlation exists between the constraint stiffness of spacer-damper connecting nodes and conductor galloping amplitude: the stronger the nodal constraint, the greater the resulting galloping amplitude. Consequently, in power transmission line engineering practice, prioritizing hinged spacer-damper connections with reduced constraints effectively mitigates galloping amplitude of ice-coated conductors, significantly enhancing both structural integrity and operational safety of transmission systems under severe weather conditions.

## 5. Conclusions

This paper proposes a three-dimensional aerodynamic numerical simulation method incorporating spanwise length effects and investigates the galloping response of ice-coated transmission conductors. Focus was placed on analyzing the impact of spanwise length on aerodynamic force calculation accuracy, elucidating the influence of torsional stiffness on conductor galloping, and proposing a transmission conductor modeling method that employs cable elements and incorporates torsional degrees of freedom. The principal findings are summarized as follows:

(1) The proposed three-dimensional aerodynamic numerical simulation method allows for an integrated consideration of the effects of near-wall mesh density and spanwise length on the aerodynamic coefficients of ice-coated conductors, which significantly enhances the computational accuracy of aerodynamic forces during galloping and thereby provides a more reliable foundation for galloping-resistant design.
(2) Under combined wind and ice loading, the galloping amplitude of quad-bundled conductors is observed to be significantly greater than that of twin-bundled conductors. Spacer bars substantially reduce torsional displacement by restricting conductor rotation.
(3) Research indicates that the constraint stiffness of spacer connection joints exhibits a significant positive correlation with conductor galloping amplitude: greater constraint leads to larger galloping amplitudes. Therefore, in transmission line engineering practice, the connection method using hinged spacers that provide less torsional restraint should be prioritized. This approach can effectively reduce the galloping amplitude of ice-coated conductors and significantly enhance the structural integrity and transmission safety of the line under adverse weather conditions.

## Supporting information

S1 Data Supporting Data. (RAR)
